# Supplementary material for: Genetic impacts on DNA methylation help elucidate regulatory genomic processes
Source: Genome Biol. 2023 Jul 31;24:176. doi: 10.1186/s13059-023-03011-x (PMC10391992; doi:10.1186/s13059-023-03011-x)
Supplement: Supplementary file 7 — Additional file 7. Review history. [file 13059_2023_3011_MOESM7_ESM.docx]

Review History

**First round of review**

**Reviewer 1**

**Were you able to assess all statistics in the manuscript, including the appropriateness of statistical tests used?**

Yes, and I have assessed the statistics in my report.

**Comments to author:**

The paper by Villicana et al. summarises a set of meta analyses across three UK based EPIC datasets to unravel the extend, distribution and functional annotation of meQTLs. EPIC arrays are the most used platforms for large scale epigenetic association studies. In this respect the paper creates a highly useful, extensive repository of meQTL calls across European populations. The authors observe that about 34% of all 724.000 CpGs informative on the array are linked to cis-meQTL effects (98%). The authors perform a number of well conducted cis- and trans- chromosomal analyses with convincing statistical testing to show the robustness of their findings.

They find a significant number CpGs on EPIC arrays to be linked to cis meQTLs. Furthermore they also describe interesting cis and trans links to eQTLs. Finally they also report that the meQTL catalogue can be used to rationalise on GWAS hits by analysing public datasets across 56 traits. Overall their work summarises a thorough and deep study showing some interesting cis and trans meQTL findings. The catalogue of their data will be an important resource for the interpretation of the widely used EPIC arrays in association studies.

Some critical points:

Particularly in the results part the text needs some clarification and revision. Concluding sentences at the end of individual chapters within the results section are convoluted and sometimes difficult to comprehend as they combine summaries and interpretation with many references to (mostly own) previous work and interpretations.

Particularly the findings and interpretations of trans-meQTLs should be considered and discussed with greater caution - the author mention that the effects were very dataset dependent and are hence much more batch effect drive as compared to robust cis effect.

Overall analyses of cell type specific effects are missing.

The validation of meQTLs using MeDIP data require a better explanation - MeDIP based enrichment quantification is difficult if not impossible and hence validation can only be qualitative in relation to EPIC data. It remains unclear why the 10 CpGs could not have been validated by deep sequencing including allelic information ( or even cell type effects) as shown in Scherer et al. 2021.

In the discussion references are missing (line 432) with respect to previous work. In general the authors should give more credit to important previous contributions to the field such as https://doi.org/10.1183/13993003.02079-2019, https://doi.org/10.1016/j.ajhg.2018.09.007, https://doi.org/10.3389/fgene.2019.01021, https://doi.org/10.1038/s41467-018-03209-9.

**Reviewer 2**

**Were you able to assess all statistics in the manuscript, including the appropriateness of statistical tests used?**

Yes, and I have assessed the statistics in my report.

**Comments to author:**

Thank you for the opportunity to review the manuscript by Villicana et al.. The authors present an clearly written study on the "Genetic impacts on DNA methylation help elucidate regulatory genomic processes" that establishes a rich resource that will be utilized by many future studies. They conducted genetic associations studies of CpG methylation sites in cis and trans based on the current generation EPIC arrays, compared findings to earlier studies based on 450k arrays, and linked associations to 56 human traits via colocalization analyses. The identified meQTLs were characterized by a host of well thought through downstream analyses. I have only a few suggestions how the manuscript could be improved.

- Discovery design: Given the >10x larger sample size in GoDMC for 450k sites, did you consider restricting your analysis on 450k sites to a replication analysis? This would increase the power on the legacy sites dramatically and also relax the threshold for the EPIC-only analyses as the discovery part of the paper would only consider half the CpG sites. However, this would limit the comparisons between legacy and new sites you are currently including in the paper. This might also affect some of the other downstream analyses as array design of 450k and EPIC arrays is not random.

- lines 112ff: Given the differences in heritability by genomic annotations it would be nice to see the comparison of 450k legacy sites with EPIC-only sites stratified by genomic annotations. This would allow to support or reject the hypothesis in lines 116ff.

- Line 439: "Intra-chromosomal trans -meQTLs appear to be 'long-range' cis -meQTLs, as the vast majority are located within 5 Mbp from the target CpG." This hypothesis could further be explored by the incorporation of topologically associating domains (TADs) estimated from Hi-C data. If the hypothesis holds the long-range cis-meQTLs should be situated in larger TADs.

- For the comparison of the power of past 450k meQTL efforts, the few existing EPIC efforts and your study, it would be helpful to include the samples size of the largest meQTL analyses (current ref 10 / Min et al?) in the introduction.

- Line 141: Consider switching to "…2,281 (43.7% of trans-meQTL SNPs; 0.9% of cis-meQTL SNPs)…" which allows for a direct comparison with the 33.7% and 0.7%.

- Line 148: Consider rephrasing the explained variance sentence, which currently could be read as 7.6% + 11.5% of the overall methylation variance in all CpGs.

**Authors Response**

**Point-by-point responses to the reviewers’ comments:**

Reviewer #1:

The paper by Villicana et al. summarises a set of meta analyses across three UK based EPIC datasets to unravel the extend, distribution and functional annotation of meQTLs. EPIC arrays are the most used platforms for large scale epigenetic association studies. In this respect the paper creates a highly useful, extensive repository of meQTL calls across European populations. The authors observe that about 34% of all 724.000 CpGs informative on the array are linked to cis-meQTL effects (98%). The authors perform a number of well conducted cis- and trans- chromosomal analyses with convincing statistical testing to show the robustness of their findings.

They find a significant number CpGs on EPIC arrays to be linked to cis meQTLs. Furthermore they also describe interesting cis and trans links to eQTLs. Finally they also report that the meQTL catalogue can be used to rationalise on GWAS hits by analysing public datasets across 56 traits. Overall their work summarises a thorough and deep study showing some interesting cis and trans meQTL findings. The catalogue of their data will be an important resource for the interpretation of the widely used EPIC arrays in association studies.

Some critical points:

Particularly in the results part the text needs some clarification and revision. Concluding sentences at the end of individual chapters within the results section are convoluted and sometimes difficult to comprehend as they combine summaries and interpretation with many references to (mostly own) previous work and interpretations.

*Response: We have re-written parts of the Results section of the manuscript to improve clarity. We also include additional references not published by our group – please see response to Reviewer 1 comment 5 below.*

*Specific changes to text in the Results are included in subsections on:*

*- Heritability (page 3 lines 32–42): “When stratifying … previously reported 450K heritability estimates across genomic annotations (Van Dongen et al. 2016).”*

*- meQTLs (page 4, lines 24–33, 37): “We carried out sensitivity analysis... which is a significant difference (Supplementary note) … However, …”; (page 5 lines 15–3): “Overall, meQTLs explain 14.2% … DNA methylation heritability.”*

*- Genomic annotations (page 6 lines 19–22): “The results remain consistent … distance to target CpGs (Fig. 4b and Supplementary table 8).”*

*- Functional integration (page 7 line 36–38): “SMR analysis with trans-meQTLs … affecting 709 CpGs and 782 genes”; (page 7 lines 45–47): “Our findings are in line … in the context of DNA repair”; (page 8 lines 17–21): “These results could reflect a genetic mechanism of transmeQTL effects … (Fig 5c).”; (page 8 lines 30–44): “We carried out two additional functional integration of meQTLs… within TADs.”; (page 9 lines 6–8): “Therefore, these results support the hypothesis … at multiple genomic regions.”*

*- Highly regulated CpGs (page 9 lines 31–32): “All these CpGs are located on chromosomal region 8p23.1 …”; (page 10 line 5): “However, the very high genetic diversity …”*

*- GWAS co-localisation (page 10 lines 34–36): “‘Growth and ageing’ … 598 unique CpGsites”; (page 11 lines 30–31): “Therefore, we hypothesise that the action of rs7187776 is through a cis-meQTL-mediation mechanism.”*

Particularly the findings and interpretations of trans-meQTLs should be considered and discussed with greater caution - the author mention that the effects were very dataset dependent and are hence much more batch effect drive as compared to robust cis effect.

*Response: We agree with the Reviewer overall. On the one hand we detect a smaller proportion of CpGs with trans-meQTLs compared to previous studies and importantly, our transmeQTL results show more heterogeneity across studies than our cis-meQTL results. On the other hand, our findings for functional enrichments and depletions for CpGs with trans-meQTLs and for trans-meQTL SNPs are very consistent with previous studies, which is very reassuring. Therefore, we keep these results, and as suggested by the Reviewer—we are now more cautious with interpretations about trans-meQTLs.*

*We explicitly mention this limitation in the Results section (page 4 lines 27–31): “The strength of the associations was stronger for trans SNP-CpG pairs than for cis SNPCpG pairs, although this is an expected result due to the difference in P-value thresholds for cis and trans associations (Supplementary note). On the other hand, trans effects were more heterogeneous across samples compared to cis effects, and therefore the reported trans effects should be interpreted with caution.”*

*We also mention this limitation in the Discussion (page 12 lines 12–16): “This likely represents lower reproducibility of trans signals, which may be more likely subject to cohort specific differences, batch effects, or may potentially represent biological factors. Therefore, the reported trans-meQTL results should be interpreted with caution and validated in future studies.”*

Overall analyses of cell type specific effects are missing.

*Response: To address the Reviewer’s comment we have now carried out additional genome-wide analyses of cell type-specific meQTLs. We now include meQTL analysis for CD4+ T cells and monocytes. Briefly, we observe a moderate number of signals detected across the entire EPIC panel, and a small number of CpGs have simultaneous cell-specific and whole blood meQTLs effects. Manuscript changes related to this comment include:*

*- Methods, new subsection ‘Cell type specific effects’ (pages 16–17 lines 35–2).*

*- Results (page 5 lines 4–13), where we state: “We also explored evidence for cell type-specific meQTL effects. These analyses considered only cis-meQTLs effects specific to CD4+ T cells (mean ratio = 0.199, sd = 0.073 across samples) and monocytes (mean ratio = 0.05, sd = 0.026 across samples). We observed that 8.9% of all CpGs had cis-meQTL effects specific for either CD4+ T cells or monocytes (P ≤ 2.21×10–4 ; see Supplementary note and Supplementary table 2). Our cell-specific results replicate a proportion of previously reported cell-specific meQTLs in CD4+ T cells (17.7%) and in monocytes (17.3%) (Chen et al., 2016). Of the CpGs that had cis-meQTL SNPs in whole blood, 1.1% also showed evidence for cell-specific meQTL effects (P ≤ 2.21×10–4 ), suggesting that the majority of genetic effects that we detect on CpGs in whole blood are stable across different blood cell types (Supplementary note).”*

*- Discussion (page 13 lines 5–9): “We undertook cell-specific analysis and observed that the majority of whole blood meQTLs do not show evidence for cell-specific effects. However, we did not comprehensively explore cell-population specific meQTL effects and restrict our analysis to two cell types with modest to moderate proportions in our whole blood data.”*

*- The full results for the new cell-specific meQTL analyses are described in section 1.6 of the Supplementary note.*

The validation of meQTLs using MeDIP data require a better explanation - MeDIP based enrichment quantification is difficult if not impossible and hence validation can only be qualitative in relation to EPIC data. It remains unclear why the 10 CpGs could not have been validated by deep sequencing including allelic information ( or even cell type effects) as shown in Scherer et al. 2021.

*Response: We thank the Reviewer for raising this point, and agree that higher-resolution technology would be useful for validation. However, our manuscript did not generate new MeDIP-seq data, but rather used previously published MeDIP-seq profiles for validation of targeted signals. We now update the text to clarify this in the Results (page 5 line 27–28):*

*“We pursued replication of meQTL effects at selected CpGs using previously published MeDIPseq data …”*

*We also agree with the Reviewer that this MeDIP-seq approach to replication is more ‘qualitative’. We now update the Discussion to incorporate this limitation (page 13 lines 11–14):*

*“Third, we carried out validation of all legacy 450K signals in the GoDMC dataset, and pursued replication at targeted novel EPIC-specific sites. The resolution of MeDIP-seq methylation data (500 bp) is lower compared to EPIC data and therefore presents a more qualitative replication approach.”*

*Overall, we include the MeDIP-seq replication component—in line with recent meQTL reports that also used MeDIP-seq validation of Illumina 450K meQTLs (e.g. Hawe et al., 2022)—because we think it important to highlight that target meQTL signals can validate across independent methylation profiling technologies.*

In the discussion references are missing (line 432) with respect to previous work. In general the authors should give more credit to important previous contributions to the field such as https://doi.org/10.1183/13993003.02079-2019, https://doi.org/10.1016/j.ajhg.2018.09.007, https://doi.org/10.3389/fgene.2019.01021, <https://doi.org/10.1038/s41467-018-03209-9>.

*Response: We thank the Reviewer for pointing this out. We now include additional references throughout the manuscript in the Introduction, Results and Discussion. We include altogether 7 new references in the revised version of the paper, as well as multiple citations of new aspects of references that were previously included in the manuscript.*

*Examples of updated text include:*

*- Introduction (page 2 lines 24–26): “Another recent analysis … (Hawe et al. 2022)”*

*- Results (page 6 line 29): “… genetic variant could prevent protein binding and alter methylation of the surrounding loci (Banovich et al., 2014; Do et al., 2016)”*

*- Results (page 6 lines 15–16): “Overall, we observe robust evidence for co-localisation between cis-meQTLs and eQTLs, which is in line with previous findings (Hannon et al., 2018; Pierce et al., 2018).”*

*- Results (page 7 line 43): “‘eQTL-mediation mechanism’ from Villicana & Bell (2021), also proposed by Huan et al. (2019)”*

*- Results (page 7 lines 35–36): “In addition, the results of GO enrichments also replicate findings from previous studies (Hawe et al., 2022, Huan et al. 2019).”*

*- Results (page 8 line 33–34) “… some intra-chromosomal trans-meQTLs may act as ‘longrange’ cis-meQTLs (Hawe et al., 2022, Huan et al., 2019) that TADs bring into physical proximity (Villicana et al. 2021, Bonder et al. 2017).”*

*- Discussion (page 12 lines 5–9, 20): “Our cis results are in line with previous studies on the Illumina EPIC and 450K, in terms of proportion of sites, distance to target, allele frequency, and genomic annotations (Min et al., 2021; Huan et al., 2019; Husquin et al., 2018). The proportion of trans signals that we detected is somewhat lower than previous studies (Min et al., 2021; Huan et al., 2019, Hannon et al., 2018).”*

*- Discussion, comparison with previous studies (page 12, lines 44–47): “Our work is consistent with and extends previous efforts, both disease-specific (Zhao et al., 2019; Kim et al. 2020) and multi-traits (Hannon et al. 2018; Wu et al. 2018), that integrate different molecular data at the genome-wide level to provide new insights into disease processes and biological pathways.”*

Reviewer #2:

Thank you for the opportunity to review the manuscript by Villicana et al.. The authors present an clearly written study on the "Genetic impacts on DNA methylation help elucidate regulatory genomic processes" that establishes a rich resource that will be utilized by many future studies. They conducted genetic associations studies of CpG methylation sites in cis and trans based on the current generation EPIC arrays, compared findings to earlier studies based on 450k arrays, and linked associations to 56 human traits via colocalization analyses. The identified meQTLs were characterized by a host of well thought through downstream analyses. I have only a few suggestions how the manuscript could be improved.

- Discovery design: Given the >10x larger sample size in GoDMC for 450k sites, did you consider restricting your analysis on 450k sites to a replication analysis? This would increase the power on the legacy sites dramatically and also relax the threshold for the EPIC-only analyses as the discovery part of the paper would only consider half the CpG sites. However, this would limit the comparisons between legacy and new sites you are currently including in the paper. This might also affect some of the other downstream analyses as array design of 450k and EPIC arrays is not random.

*Response: We thank the Reviewer for this suggestion. We have now addressed this as a sensitivity analysis, where we split EPIC probes in two sets—legacy probes and EPIC-specific probes. We then carry out analyses for each set separately, estimating genome-wide discovery meQTL P-values thresholds separately. Interestingly, we observe that these two new thresholds (for the 450K legacy probes set Pcis ≤ 2.23 × 10−4 and Ptrans ≤ 4.87 × 10−9 ; for the novel EPIC-only probes set Pcis ≤ 2.18 × 10−4 and Ptrans ≤ 1.84 × 10−9 ) were almost equal to that used in the main meQTL analysis (Pcis ≤ 2.21x10-4 , Ptrans ≤ 3.35x10-9 ).*

*To address this comment, we have now made the following changes to the manuscript:*

*- Results (page 4 lines 24–27):*

*“We carried sensitivity analysis by splitting the CpGs into two sets, 450K legacy probes and EPIC-specific probes, and repeating the meQTL discovery process, and found that the resulting proportions of meQTLs reported remained very similar (Supplementary note).”*

*- Methods (page 16 lines 20–23):*

*“As a sensitivity analysis, we also estimated the threshold P-values by dividing the EPIC CpGs into two sets (legacy 450K probes only and the novel EPIC-only probes).”*

*- The full details of the approach are outlined in section 1.12 of the Supplementary note.*

- lines 112ff: Given the differences in heritability by genomic annotations it would be nice to see the comparison of 450k legacy sites with EPIC-only sites stratified by genomic annotations. This would allow to support or reject the hypothesis in lines 116ff.

*Response: We have now added the suggested comparison in the manuscript. We observe that heritability patterns are consistent between the different types of probes (Supplementary Figure 1b, c). This observation is in support of the claim that higher heritabilities on the EPIC array compared to the 450K are likely due to the increased enhancer coverage. Additionally, novel EPIC probes annotated in promoters on average have higher heritabilities compared to legacy 450K CpGs also annotated in promoters.*

*To address this comment, we have made the following changes to the manuscript:*

*- Results (page 3 lines 39–42), where we state:*

*“Overall, the heritability patterns of the genomic annotations are consistent between EPIConly probes and 450K legacy probes (Supplementary fig. 1b-c), and are broadly in line with previously reported 450K heritability estimates across genomic annotations (Van Dongen et al., 2016).”*

*- We have also updated Supplementary fig. 1.*

- Line 439: "Intra-chromosomal trans -meQTLs appear to be 'long-range' cis -meQTLs, as the vast majority are located within 5 Mbp from the target CpG." This hypothesis could further be explored by the incorporation of topologically associating domains (TADs) estimated from Hi-C data. If the hypothesis holds the long-range cis-meQTLs should be situated in larger TADs.

*Response: We thank the Reviewer for this comment. As suggested, we extended our analysis and also included additional Hi-C data to better annotate TADs. We used domains predicted by the 3D Genome Browser (Wang et al., 2018) for the GM12878 cell line (data from Rao et al., 2014, and Lieberman et al., 2009), and extended the analyses to also include TADs in thymus and spleen (data from Schmitt et al., 2016, and Leung et al. 2015). As predicted by the Reviewer, we found a significant difference in TAD sizes. TADs containing intra-chromosomal trans-meQTLs were larger than those containing cis-meQTLs, supporting our statement that intra-chromosomal trans-meQTL may act like ‘long-range’ cis-meQTLs.*

To address this comment, we now make the following changes to the manuscript:

*- Methods (page 18, lines 18–28) “We incorporated 3D genomic annotations in the meQTL functional annotations by using previously published data from Hi-C experiments in lymphoblastoid cell line GM12878 (Rao et al., 2014, Lieberman et. al., 2009). We also considered additional Hi-C data across multiple relevant cells and tissues, including in GM12878, in spleen (Schmitt et al., 2016) and thymus (Leung et al. 2015). TADs from these datasets were the generated in and obtained from the 3D Genome Browser (Wang et al., 2018). We estimated the percentage of CpGs where the target CpG and its most associated meQTL fell within the same TAD. This analysis was carried out for all CpGs with cis-meQTLs and for all CpGs with intra-chromosomal trans-meQTLs. The estimation was performed for the GM12878 Hi-C data alone, as well as for TADs estimated from multiple cells and tissues. Further details and results are provided in the Supplementary note”.*

*- Results (page 8 lines 32–44), includes a summary of the most relevant results:*

*“The rationale behind this analysis was that some intra-chromosomal trans-meQTLs may act as ‘long-range’ cis-meQTLs (Hawe et al., 2022, Huan et al., 2019) that TADs bring into physical proximity (Villicana et al., 2021, Bonder et al., 2017). We integrated our meQTL results with TADs predicted from multiple-tissue Hi-C experiments (Wang et al., 2018, Rao et al., 2014, Lieberman et al., 2009, Schmitt et al., 2016, Leung et al., 2015). We found that 36.5% of CpGs with intra-chromosomal trans-meQTLs share the same TAD with their most associated meQTL. In comparison, 17.1% of CpGs with cis-meQTLs share the same TAD with their most associated meQTL. Furthermore, TADs containing intra-chromosomal transmeQTL associations are significantly larger than TADs with cis-meQTL associations (mean TAD sizecis= 1.2 Mbp; mean TAD sizetrans = 3.4 Mbp; P < 2.54x10-13), which supports our hypothesis that TADs may bring trans-meQTLs into physical proximity with their target CpG (Supplementary note). In summary, our results are consistent with the hypothesis that some intra-chromosomal trans-meQTLs may act as ‘long-range’ cis-meQTLs within TADs.”*

*- The full Methods and Results for this analysis are now described in section 1.9 of the Supplementary note.*

- For the comparison of the power of past 450k meQTL efforts, the few existing EPIC efforts and your study, it would be helpful to include the samples size of the largest meQTL analyses (current ref 10 / Min et al?) in the introduction.

*Response: We thank the Reviewer for this suggestion. We now explicitly mention the sample size of the GoDMC study, and also refer to the recent Hawe et al. 2022 450K meQTL study (page 2 lines 20–26), where we state:*

*“A recent large-scale study in 27,750 European samples estimated that DNA methylation levels at up to 45% of CpGs in the blood 450K methylome are associated with meQTL SNPs {Min2021}, which are in turn more likely to be GWAS signals than expected by chance. Another recent analysis of 3,799 European and 3,195 South Asian samples further explored trans-ancestry effects, and confirmed multiple links between meQTLs and phenotype variation (Hawe et al., 2022).”*

- Line 141: Consider switching to "…2,281 (43.7% of trans-meQTL SNPs; 0.9% of cis-meQTL SNPs)…" which allows for a direct comparison with the 33.7% and 0.7%.

*Response: We thank the reviewer for this comment. We rephrased this as suggested by the Reviewer (page 4 line 19), where we state:*

*“0.9% of CpGs with cis-meQTL; 43.7% of CpGs with trans-meQTL …”*

- Line 148: Consider rephrasing the explained variance sentence, which currently could be read as 7.6% + 11.5% of the overall methylation variance in all CpGs.

*Response: We thank the Reviewer for pointing this out. We have addressed this comment by revising the text. The updated text now reads (page 4 lines 31–34): “We estimated that, on average, a cis-meQTL explains 7.6% of the methylation variance of its associated CpG, while a trans-meQTL explains 11.5%, which is a significant difference (Supplementary note).”*

**Second round of review**

**Reviewer 2**

Thank you for the thorough revision that addresses all of my previous comments. Very nice to see that the TADs worked out.
